# Why so low? An unusual case of myositis in a child

**DOI:** 10.1186/s12969-023-00816-9

**Published:** 2023-04-18

**Authors:** Meagan E. Chriswell, Robert C. Fuhlbrigge, Mark A. Lovell, Matthew Monson, Jessica L. Bloom

**Affiliations:** 1 School of Medicine, University of Colorado Anschutz Medical Campus, Aurora, CO USA; 2 Department of Pediatrics, Section of Pediatric Rheumatology, University of Colorado Anschutz Medical Campus, Aurora, CO USA; 3 Department of Pathology and Laboratory Services, University of Colorado Anschutz Medical Campus, Aurora, CO USA; 4 Department of Radiology, University of Colorado Anschutz Medical Campus, Aurora, CO USA

**Keywords:** Sarcoidosis, Myositis, Granuloma, Granulomatous myositis, Pediatric, Muscle biopsy, Orbital myositis, Rare disease

## Abstract

**Background:**

Sarcoidosis is characterized by non-caseating epithelioid granulomas in various tissues throughout the body, most commonly the lung. Non-caseating granulomas may be seen in skeletal muscle, though typically asymptomatic and under-recognized. While rare in children, there is a need to better characterize the disease and its management. Here we present a 12-year-old female with bilateral calf pain who was ultimately found to have sarcoid myositis.

**Case Presentation:**

A 12-year-old female presented to rheumatology with significantly elevated inflammatory markers and isolated lower leg pain. MRI of the distal lower extremities demonstrated extensive bilateral myositis with active inflammation, atrophy, and to a lesser extent fasciitis. This distribution of myositis in a child garnered a broad differential requiring a systematic evaluation. Ultimately, muscle biopsy revealed non-caseating granulomatous myositis with perivascular inflammation, extensive muscle fibrosis, and fatty replacement of the muscle with a CD4+ T cell predominant, lymphohistiocytic infiltrate consistent with sarcoidosis. Review of histopathology from age 6 of an extraconal mass resected from her right superior rectus muscle further confirmed the diagnosis. She had no other clinical symptoms or findings of sarcoidosis. The patient improved significantly with methotrexate and prednisone, though flared again after self-discontinuation of medications and was subsequently lost to follow-up.

**Conclusion:**

This is the second reported case of granulomatous myositis associated with sarcoidosis in a pediatric patient, and the first to present with a chief complaint of leg pain. Increased knowledge of pediatric sarcoid myositis within the medical community will enhance recognition of the disease, improve the evaluation of lower leg myositis, and advance outcomes for this vulnerable population.

## Introduction

Sarcoidosis is a multisystem disease characterized by the development of non-caseating epithelioid granulomas in various tissues throughout the body, especially the lung. While sarcoidosis can develop in children, it is rare with a prevalence of approximately 0.6–1.2 per 100,000 children and an incidence of 0.29 per 100,000 children per year. To date, only three retrospective studies have characterized pediatric sarcoidosis [1], indicating a need to better characterize this rare disease and its management.

The distribution of sarcoidosis in childhood is bimodal with a triad of rash, uveitis, and arthritis more common before four years of age [2] and a multisystem presentation resembling adult sarcoidosis (pulmonary infiltrations, hilar lymphadenopathy, and occasionally ocular or cutaneous manifestations) more common around adolescence [2]. First-line management for pediatric sarcoidosis includes oral glucocorticoids but the ideal steroid-sparing option is unknown [3]. Methotrexate, azathioprine, cyclophosphamide, chlorambucil, anti-cytokine biologics and cyclosporine have been utilized in adults with variable efficacy [4, 5].

Though underrecognized, non-caseating granulomas in sarcoidosis may also occur in skeletal muscle [6–9]. In fact, 50–80% of patients with sarcoidosis are suspected to have asymptomatic muscle involvement [9–11] while 0.5–2.3% have symptomatic myositis [12] (muscle pain and/or weakness [12, 13]), such that sarcoidosis is the most common cause of granulomatous myositis [7, 12]. Other causes of granulomatous myositis include Crohn’s disease, tuberculosis, brucellosis, lymphoma, syphilis, thymoma/myasthenia gravis, primary biliary cirrhosis, granulomatosis with polyangiitis, rheumatoid arthritis, and systemic sclerosis [14]. Diagnosis is confirmed on muscle biopsy in the absence of any other explanation, such as infection, malignancy, or other inflammatory myopathies [15–19]. Despite the frequency of myositis in sarcoidosis, there is only one pediatric report in the literature.

Here, we report a pediatric patient presenting with severe sarcoid myositis of both lower legs and a periorbital muscle without other signs or symptoms of sarcoidosis. We aim to increase awareness of this underrecognized disease presentation and its response to therapeutic interventions as well as the unique chief complaint of isolated lower leg myositis.

## Case Presentation

A six-year old female presented to the emergency department with a one month history of progressive right eye swelling. At this time, laboratory findings included ESR 120 mm/hr, CRP 7.2 mg/dL, HGB 10.1 g/dL, PLT 504 × 10^9^/L, normal WBC and differential, negative angiotensin converting enzyme, negative anti-serine-protease 3 antibody, and negative anti-myeloperoxidase antibody (Table 1). Brain MRI demonstrated a T2 hyperintense, well-marginated extraconal mass centered in the right superior rectus muscle. Subsequent periorbital biopsy findings were described as consistent with eosinophilic angiocentric fibrosis. She had no notable sinusitis on imaging nor cardiorespiratory symptoms. Ophthalmologic exam showed no intraorbital inflammation. She improved with 1 mg/kg of prednisone twice daily and was successfully weaned off by one year later with mild residual upper eyelid swelling and ptosis.

**Table 1:** Laboratory test results over time

Laboratory Test (normal range)	Visit 1	Visit 2	Visit 3	Visit 4	Visit 5
	Age 6	Age 12	Age 15	Age 15 (2 months after starting glucocorticoids; methotrexate prescribed at visit)	Age 16 (1 month after stopping medications)
Erythrocyte Sedimentation Rate (0–29 mm/hr)	120 (H)	112 (H)	107 (H)	28 (H)	84 (H)
C-Reactive Protein (0-0.9 mg/dL)	7.2 (H)	7.0 (H)	7.5 (H)	1.6 (H)	14.6 (H)
IgG (700–1600 mg/dL)	-	4040 (H)	3240 (H)	2597 (H)	2540 (H)
White Blood Cell Count (5-10^3^/mcL)	7.7	9.28 (H)	11 (H)	10.9 (H)	10.46 (H)
Hemoglobin (12.1–15.1 g/dL)	10.1 (L)	9.1 (L)	9.1 (L)	10.6 (L)	9.4 (L)
Platelets (150–400 × 10^9^/L)	504 (H)	603 (H)	664 (H)	527 (H)	617 (H)
Mean Corpuscular Volume (81–88 fL)	67.8 (L)	65.6 (L)	64.9 (L)	68.8 (L)	64.9 (L)
Creatine Kinase (22–198 U/L)	26	26	30	278 (H)	38
Lactate Dehydrogenase (varies, U/L)	378 (L)	350 (L)	276 (L)	319 (L)	-
Aspartate Aminotransferase (5–30 U/L)	-	39	49 (H)	23	21
Alanine Aminotransferase (10–35 U/L)	-	15	19	14	9 (L)
Complement C3 (80–160 mg/dL)	-	191 (H)	181 (H)	-	-
Complement C4 (12–42 mg/dL)		61.9 (H)	68.4 (H)		
Lupus Anti-Coagulant (≤1.21 ratio)	-	(Negative in 2018)	1.28 (H)	-	-
d-Dimer (< 0.5 µg/mL)	-	-	0.92 (H)	< 0.27 (H)	0.82 (H)
Partial Thromboplastin Time (25–35 s)	34	-	35.5 (H)	35.5 (H)	43.9 (H)
Prothrombin Time (11-13.5 s)	15.8 (H)	-	15.1 (H)	-	15.1 (H)
International Normalized Ratio (< 1.1)	1.21	-	1.2	-	1.21
Additional labs checked during her course	Negative: antinuclear antibody profile, anti-β-2 glycoprotein-1 immunoglobulin G, anti-β-2 glycoprotein-1 immunoglobulin M, anti-cardiolipin immunoglobulin G, anti-cardiolipin immunoglobulin M, anti-myeloperoxidase antibody, anti-serine protease 3 antibody, myositis antibody panel, tissue transglutaminase immunoglobulin A Normal: aldolase, angiotensin converting enzyme, eosinophil counts, lysozyme, serum calcium, uric acid, urinalysis with microscopy, urine protein to creatinine ratio, von Willebrand factor antigen				

**Fig. 1:**
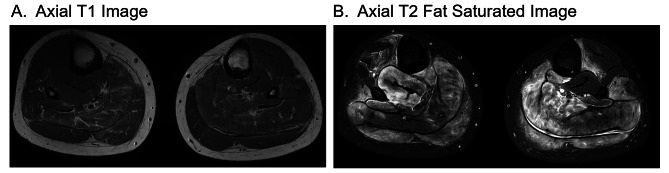
Inflammation, atrophy, and fasciitis on lower limb MRI. An axial T1 image (**A**) demonstrates increased intramuscular fat signal bilaterally consistent with atrophy. An axial T2 fat saturated image (**B**) demonstrates diffuse muscular edema in all four compartments

Six years later (age 12), she presented to pediatric rheumatology clinic with a two month history of bilateral calf pain and tightness with difficulty ambulating. She had a normal review of systems including no fever, fatigue, night sweats, or recent travel. Recent ophthalmologic follow-up exam showed no inflammation. She did not take medications and had an unremarkable family history. Exam revealed tender, swollen calves with limited ankle range of motion and strength due to pain and tightness. She had an antalgic gait. She had no hepatosplenomegaly, arthritis, rash, or abnormal cardiopulmonary findings on exam. Labs at this visit demonstrated ESR 112 mm/hr, CRP 7 mg/dL, HGB 9.1 g/dL, and MCV 65.6 with normal muscle markers and mildly elevated complement (Table 1). She had an IgG of 4040 mg/dL (503–1719), determined to be a polyclonal hypergammaglobulinemia. She also had a normal or negative ANA profile, anti-serine protease 3 antibody, anti-myeloperoxidase antibody, angiotensin converting enzyme, lysozyme, serum calcium, creatinine, aldolase, uric acid, T4, and TSH. MRI of the distal lower extremities demonstrated extensive bilateral myositis of the lower legs with both active inflammation and atrophy and to a lesser extent fasciitis (Fig. 1A). During this period of diagnostic assessment, the patient’s leg pains self-resolved and the patient was lost to follow-up.

The patient presented again to rheumatology three years later, at age 15, with three weeks of lower leg pain with swelling and tightness and an otherwise unremarkable history and exam. Her labs revealed ESR 107 mm/hr, CRP 7.5 mg/dL, and IgG 3240 mg/dL with a normal SPEP (predominantly IgG1). She tested positive for lupus anticoagulant (dilute Russell viper venom test) and anti-beta-2 glycoprotein-1 IgM and had an elevated d-Dimer, PTT, and PT. ANA profile and muscle enzyme levels remained negative (Table 1). Chest radiograph was normal. MRI with and without contrast revealed normal pelvis and thigh musculature but diffuse muscular edema consistent with severe myositis throughout her lower legs with associated fatty infiltration indicative of muscular atrophy (Fig. 1B).

**Fig. 2:**
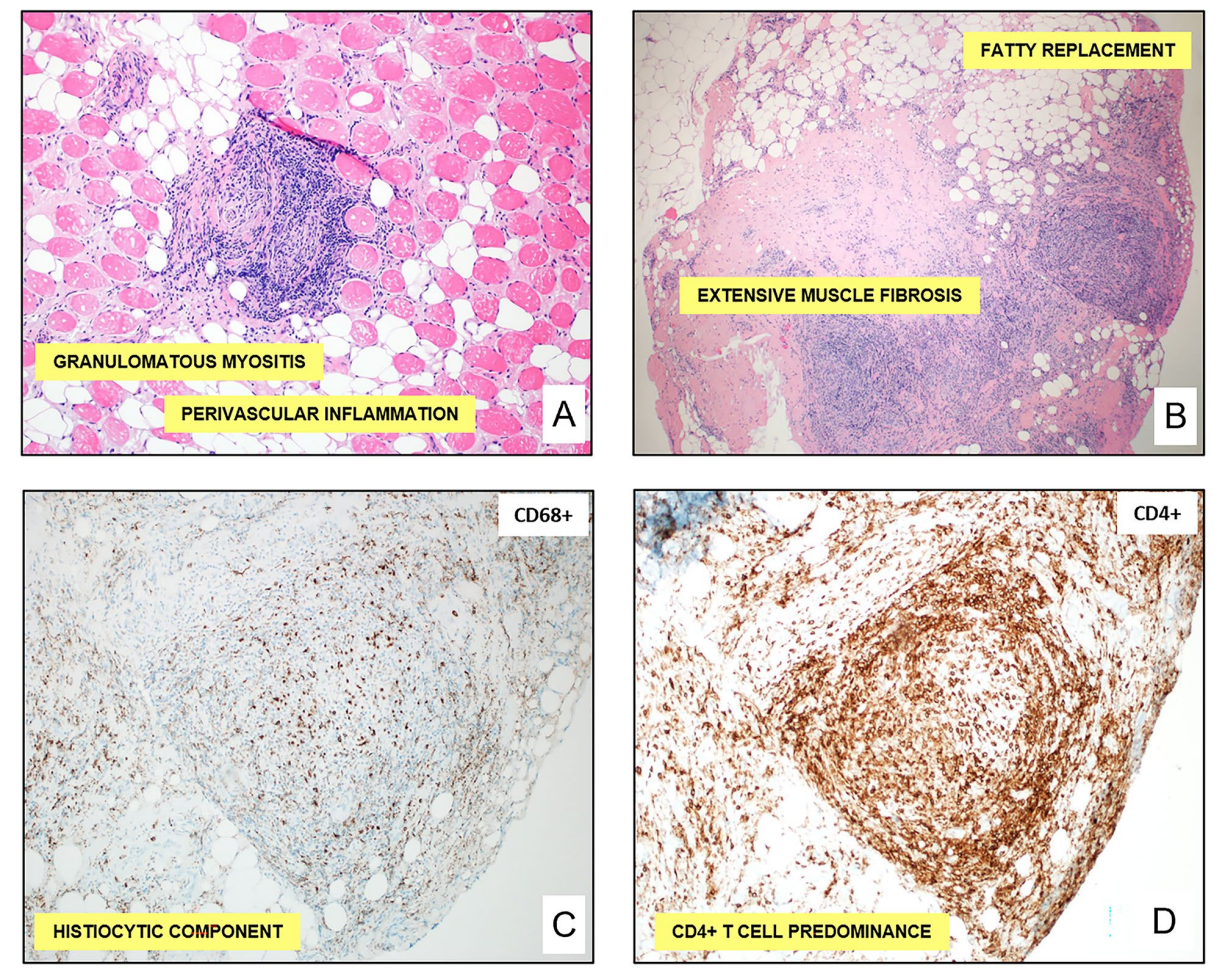
Gastrocnemius biopsy demonstrates granulomatous myositis with a histiocytic component with CD4+ T cell predominance. Left gastrocnemius biopsy revealed granulomatous myositis with perivascular inflammation (**A**, H&E, 100X) and extensive muscle fibrosis and fatty replacement (**B**, H&E, 40X). Special stains of the granulomas revealed a histiocytic component (**C**, CD68+ stain, 100X) with CD4+ T cell predominance (**D**, CD4+ stain, 100X).

A biopsy of her left gastrocnemius muscle was performed, demonstrating non-caseating granulomatous myositis with perivascular inflammation, extensive muscle fibrosis, and fatty replacement of the muscle (Fig. 2A,B). Special stains of the granulomas demonstrated a histiocytic component with CD4 + T cell predominance consistent with sarcoidosis (Fig. 2C,D). Further pathologic assessment excluded microorganisms, inclusion body myositis, and IgG4 disease.

Re-examination of her right peri-orbital biopsy from 9 years earlier also revealed a CD4 + T cell-predominant inflammatory cell infiltrate within the skeletal muscle similar to the current findings in her gastrocnemius, although no granulomas were found. She had a normal chest x-ray and no uveitis, lymphadenopathy, arthritis, or rash.

The patient was diagnosed with sarcoid myositis and prescribed 20 mg oral prednisone daily, iron (for iron deficiency), and physical therapy.

Over the next two months, she had significant improvement in her symptoms and inflammatory markers (Table 1). She began 20 mg oral methotrexate weekly with further resolution of symptoms (apart from chronic muscle tightness) when seen 4 months later on 10 mg prednisone. A few months after that visit, she self-discontinued her medications and experienced return of symptoms and lab abnormalities after one month (Table 1). Chest radiograph remained normal. Ultrasound with venous doppler of her more tender left lower extremity was reassuring against clot. Abdominal and pelvic CT performed soon after due to a motor vehicle accident showed no intrabdominal masses or concerning pathology. She was once again lost to follow-up.

## Discussion and conclusion

This is the second reported case of granulomatous myositis associated with sarcoidosis in a pediatric patient, and the first to present with a chief complaint of leg pain. While the histopathology results eventually confirmed the diagnosis, we first considered many other diagnoses given the unusual presentation of pain and inflammation localized to bilateral calves in a child.

The differential diagnosis for myositis in children includes rheumatic diseases (e.g. juvenile dermatomyositis/polymyositis, vasculitis, eosinophilic fasciitis, systemic lupus erythematosus, or IgG4-related disease), as well as infection, malignancy, paraneoplastic syndromes, and genetic, metabolic, neurologic, or toxic myopathies. Of all these conditions, only rare distal muscular dystrophies and inclusion body myositis are known to cause isolated lower leg myositis and inclusion body myositis is almost exclusively found in adults. When present in adults, symptomatic lower leg myositis is typically unilateral and involves the gastrocnemius, vastus lateralis, and adductor muscles [20, 21]. Systemic rheumatologic diseases such as juvenile dermatomyositis and polymyositis are often more proximal in nature, while vasculitis typically includes systemic symptoms and would be unlikely to cause regional muscle inflammation isolated to symmetric regions. Infectious myopathies are also unlikely to present in symmetric areas. Concern for IgG4-related disease was raised given her prior peri-orbital inflammation; however, staining of both biopsies excluded this. Her peri-orbital muscle inflammation and granulomatous myositis of her lower extremities also raised concern for granulomatosis with polyangiitis; however, she had no signs of vasculitis on histopathology, imaging, or exam and negative anti-neutrophil cytoplasmic antibodies [22]. Lastly, the intermittent nature of her flares over the years was unusual for any of these diagnoses - though given the degree of atrophy seen, it is likely that some inflammation persisted between presentations.

As demonstrated, a diagnosis of sarcoidosis requires consideration of a multitude of diagnoses. In these diagnostic dilemmas, a thorough laboratory evaluation must assess for signs of inflammation (CBC, ESR, CRP, IgG) and muscle injury (CK, LDH, AST, and aldolase). Unfortunately, results in this patient were non-specific and her significantly elevated IgG raised concern for monoclonal gammopathy or multiple myeloma initially, though SPEP was reassuring. While she did have a positive lupus anticoagulant, this finding has low specificity and she lacked other antiphospholipid antibodies, signs and/or symptoms of thrombosis, or markers of systemic lupus erythematosus. Ultimately, tissue biopsy was deemed necessary and led to a diagnosis of granulomatous myositis. While granulomatous myositis can be seen in the context of infections (such as tuberculosis), inflammatory bowel disease, vasculitis, malignancy, thymoma, and myasthenia gravis, [23] her presentation, negative staining for infectious pathogens, and lymphohistiocytic infiltrate with CD4 + T cell predominance confirmed the diagnosis of sarcoid myositis. With this knowledge, re-evaluation of her prior periorbital biopsy further supported a diagnosis of sarcoidosis.

There are 3 clinical patterns of sarcoid myositis reported in the literature: acute inflammatory myositis with myalgia (typically presents early on and in young patients), chronic myopathy with progressive weakness (typically presents late and is the most common pattern), and nodular myopathy with palpable muscle nodules. Our patient presented with acute inflammatory myositis with myalgia that progressed toward chronic myopathy. It is important to note that markers of sarcoidosis (angiotensin converting enzyme and lysozyme) are often negative in children. Additionally, markers of muscle inflammation may be normal in sarcoid myositis, as seen in our patient.

Interestingly, our patient had no other features of sarcoidosis, apart from elevated inflammatory markers and her histopathology, despite almost a decade of disease activity affecting various muscles. This is in contrast to the one previously reported pediatric case of sarcoid myositis in which a child presented at 16 years of age with hypercalcemia and renal insufficiency and was subsequently found to have non-caseating epithelioid granulomas and calcium oxalate crystals in her skeletal muscle after abnormalities were noted on exam and imaging [24].

Little is known about the optimal treatment of sarcoidosis in children, including sarcoid myositis. While sarcoidosis typically responds to glucocorticoids, the response of sarcoid myositis to glucocorticoids is unpredictable [7, 10, 12, 15, 25, 26]. Both our patient and the previously reported pediatric patient responded well to prednisone and methotrexate with relapse upon glucocorticoid withdrawal. The 16-year-old went into remission with the addition of adalimumab while our patient was lost to follow-up. There are reports of successful treatment of adults with sarcoid myositis with glucocorticoids [10, 27] with or without methotrexate [28] as well as other immune modulating agents, including TNF-antagonists.

In summary, sarcoid myositis is an underrecognized manifestation of sarcoidosis with very little known regarding its presentation and course in children. In this patient, sarcoid presented as bilateral lower leg myositis, a rare distribution of myositis that required a broad differential diagnosis and targeted work-up. This patient also fell into the rare subgroup of patients with symptomatic muscle involvement [12]. Further characterization of sarcoid myositis in children and its management will enhance care for children with this potentially devastating disease.

## Data Availability

Data sharing is not applicable to this article as no datasets were generated or analysed for use in this case report.

## Abbreviations

ESR: Erythrocyte Sedimentation Rate
CRP: C-Reactive Protein
HGB: Hemoglobin
PLT: Platelets
WBC: White Blood Cells
MRI: Magnetic Resonance Imaging
MCV: Mean Corpuscular Volume
TSH: Thyroid Stimulating Hormone
SPEP: Serum Protein Electrophoresis
PTT: Partial Thromboplastin Time
PT: Prothrombin Time
ANA: Antinuclear Antibody
CBC: Complete Blood Count
CK: Creatinine Kinase
LDH: Lactate Dehydrogenase
AST: Aspartate Aminotransferase
TNF: Tumor Necrosis Factor
